# TLR7 F507S gain-of-function mutation presenting with early-onset SLE and hypertriglyceridemia: Response to JAK inhibition

**DOI:** 10.70962/jhi.20250196

**Published:** 2026-01-06

**Authors:** Nima Parvaneh, Hossein Karami, Leila Shahbaznejad, Zahra Rezaei, Parastoo Rostami, Mohammad Shahrooei, Vahid Ziaee

**Affiliations:** 1 Division of Allergy and Clinical Immunology, Department of Pediatrics, Tehran University of Medical Sciences, Tehran, Iran; 2 Thalassemia Research Center, Hemoglobinopathy Institute, Mazandaran University of Medical Sciences, Sari, Iran; 3 Pediatric Infectious Diseases Research Center, Communicable Diseases Institute, Mazandaran University of Medical Sciences, Sari, Iran; 4 Children’s Medical Center, Pediatrics Center of Excellence, Tehran University of Medical Sciences, Tehran, Iran; 5 Pediatric and Adolescence Multidisciplinary Epilepsy Research Center, Tehran University of Medical Sciences, Tehran, Iran; 6 Division of Endocrinology and Metabolism, Department of Pediatrics, Children’s Medical Center, Tehran University of Medical Sciences, Tehran, Iran; 7 Dr. Shahrooei Lab, Tehran, Iran; 8 Pediatric Rheumatology Research Group, Rheumatology Research Center, Tehran University of Medical Sciences, Tehran, Iran; 9 Department of Pediatrics, Tehran University of Medical Sciences, Tehran, Iran

## Abstract

We present a patient harboring the TLR7 F507S mutation who initially presented with refractory thrombocytopenia that evolved into SLE with subtle neurologic features, accompanied by hypertriglyceridemia. Both hematological and metabolic features responded to ruxolitinib, thereby expanding the phenotypic spectrum and therapeutic understanding of TLR7 GOF disease.

Toll-like receptor 7 (TLR7) is an endosomal pattern recognition receptor encoded by a gene located on the X chromosome that detects single-stranded RNA and is crucial for type I interferon production. Under normal physiological conditions, TLR7 trafficking, degradation, and signaling are strictly regulated to maintain immune homeostasis.

The clinical significance of this pathway has been elucidated through the identification of both loss-of-function (LOF) and gain-of-function (GOF) mutations. *TLR7* LOF mutations result in severe COVID-19 susceptibility due to compromised interferon responses. Conversely, *TLR7* GOF mutations induce hyperactive signaling that escapes normal regulation, leading to increased type I interferon production and autoimmunity (1). Recent studies have identified GOF mutations in *TLR7* as a monogenic etiology of systemic lupus erythematosus (SLE) and associated interferonopathies with severe neurologic involvement resembling Aicardi–Goutières syndrome (AGS) (1, 2, 3, 4).

We present a patient harboring the TLR7 F507S mutation who initially presented with refractory thrombocytopenia that evolved into SLE with subtle neurologic features, accompanied by hypertriglyceridemia, a previously unreported metabolic manifestation. Both hematological and metabolic features responded to ruxolitinib, thereby expanding the phenotypic spectrum and therapeutic understanding of TLR7 GOF disease.

A female infant born to non-consanguineous Iranian parents presented at 1 mo of age with recurrent hematochezia. Physical examination revealed pallor and irritability, with no lymphadenopathy or organomegaly. Initial laboratory investigations exhibited thrombocytopenia (platelet count: 29 × 10^9^/L) and anemia (hemoglobin: 8.9 g/dl). Bone marrow aspiration (BMA) excluded malignancy and hemophagocytosis, supporting a diagnosis of immune thrombocytopenia. Initial treatment with dexamethasone (0.6 mg/kg/day for 4 days) and intravenous immunoglobulin (IVIG, 1 g/kg) yielded transient improvement; however, frequent relapses necessitated repeated courses of therapy (Fig. 1, A and B). Notably, gastrointestinal bleeding resolved completely after the initial episode and did not recur.

**Figure 1.**
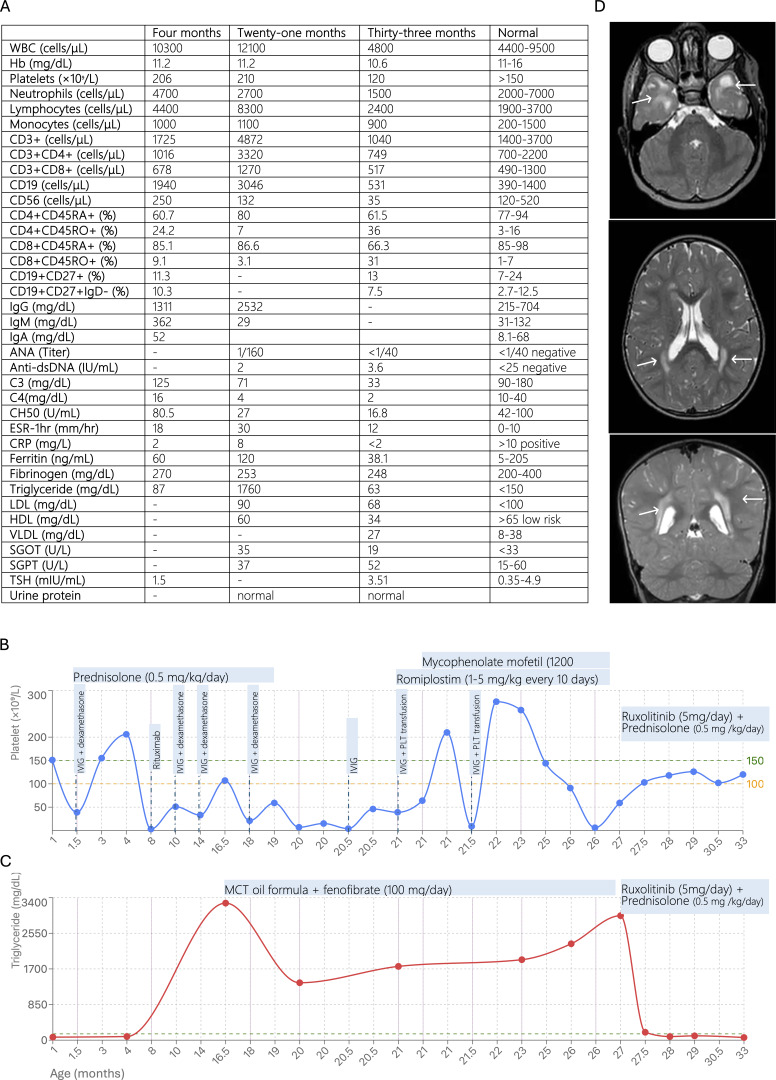
**Laboratory and radiological features of the patient with TLR7 F507S GOF disease. (A)** Summary of immunologic and metabolic evaluations. **(B)** Longitudinal platelet counts demonstrating refractory thrombocytopenia despite conventional therapy and modest response following ruxolitinib initiation. **(C)** Serum triglyceride trajectory showing severe hypertriglyceridemia (peaks at 16.5 and 27 mo) and normalization to <150 mg/dl following combination therapy with ruxolitinib and prednisolone. **(D)** T2-weighted magnetic resonance imaging images (axial and coronal) at 30 mo revealing bilateral temporal subcortical and periventricular white matter abnormalities (white arrows). ANA, antinuclear antibody. WBC, white blood cells; ANA, antinuclear antibody; Anti-dsDNA, anti-double stranded DNA antibody; ESR, erythrocyte sedimentation rate; CRP, C-reactive protein; LDL, low-density lipoprotein; HDL, high-density lipoprotein; VLDL, very low-density lipoprotein; SGOT, serum glutamic-oxaloacetic transaminase; SGPT, serum glutamic-pyruvic transaminase.

At 2.5 mo, she developed Bacillus Calmette-Guérin lymphadenitis affecting the ipsilateral axillary lymph nodes. Drainage material tested negative for acid-fact bacilli, and clinical and imaging findings showed no evidence of dissemination. She was treated with a 3-mo course of isoniazid and vitamin B6. An immunologic workup at 4 mo, including immunoglobulin levels and lymphocyte subsets, was unremarkable (Fig. 1 A). A repeat BMA at 8 mo showed increased megakaryocytes with normal morphology. The patient exhibited no fever, lymphadenopathy, or splenomegaly, and laboratory criteria for hemophagocytic lymphohistiocytosis were not met. Rituximab therapy (375 mg/m^2^) was attempted but discontinued early due to a severe allergic reaction.

At 16 mo, metabolic screening showed isolated severe hypertriglyceridemia (3,270 mg/dl), while renal, hepatic, and thyroid functions remained normal. Management with a medium-chain triglyceride formula and fenofibrate (100 mg/day) resulted in a transient decrease in triglycerides to ∼1,400 mg/dl by month 20, followed by a rebound (Fig. 1 C).

Whole-exome sequencing identified a de novo missense variant in exon three of *TLR7* (c.1520G > T; F507S), confirmed by Sanger sequencing, and absent in both parents.

By 21 mo, thrombocytopenia remained refractory to conventional therapies. The patient received IVIG, romiplostim (1–5 µg/kg every 10 days), and platelet transfusions, which provided only short-term correction of platelet counts. Concurrently, triglyceride levels rose to 1,700 mg/dl. The patient subsequently developed livedo reticularis, and rheumatologic evaluation revealed a positive antinuclear antibody titer (1:160) and significant hypocomplementemia (Fig. 1 A).

Following a diagnosis of SLE, mycophenolate mofetil (1,200 mg/m^2^) was initiated. While the rash improved, thrombocytopenia persisted (50–70 × 10^9^/L), and hypertriglyceridemia exacerbated, reaching a second peak of ∼2,900 mg/dl at 27 mo.

Combination therapy with ruxolitinib (5 mg/day) and prednisolone (0.5 mg/kg/day) was subsequently initiated. This resulted in a rapid response: by month 27.5, triglyceride levels normalized (<150 mg/dl) and remained stable through month 33. Concurrently, the patient achieved sustained but modest control of thrombocytopenia (100–120 × 10^9^/L).

The severity of the thrombocytopenia and the subsequent hypertriglyceridemia initially obscured subtle neurological problems. However, early developmental milestones were delayed. The patient sat with assistance at 9 mo, sat independently at 11–12 mo, and walked without aid at 24 mo. Language development was similarly delayed, with speech onset occurring between 18 and 24 mo.

The Denver Developmental Screening Test, performed at 33 mo, indicated delays in gross motor (consistent with an 18-mo developmental age) and fine motor skills (26 mo), which were more pronounced than delays in language (30 mo) and personal-social domains (30 mo). Neurological examination at 33 mo revealed no focal deficits. Deep tendon reflexes were 3+ and symmetrical, with normal muscle tone and no spasticity. Although the patient exhibited clumsiness while walking, gait was not abnormal. No hand or foot dominance was observed. Following the initiation of ruxolitinib, significant improvements were noted in all developmental domains, particularly in language and cognition. Brain magnetic resonance imaging revealed bilateral temporal subcortical hyperintensities and bilateral periventricular white matter changes (Fig. 1 D).

Our patient harbored the TLR7 F507S variant, a pathogenic mutation that has been previously reported and functionally validated. This mutation produces a GOF in TLR7 by altering the homodimerization interface, which in turn enhances TLR7 signaling and leads to increased nuclear factor κ B activation when stimulated with TLR7 agonists (2).

A review of all reported *TLR7* GOF mutations identifies 11 patients from 8 families, including the current case. The median age at disease onset was 8 mo (range: second day of life to mid-20s), with marked female predominance (10/11 patients, 91%), likely reflecting the severity of the phenotype in hemizygous males rather than solely the X-linked dominant inheritance pattern. Remarkably, the single affected male presented with severe early-onset disease and devastating neurological complications, suggesting that males who survive may present a severe phenotype. De novo mutations were described in 5 out of 11 cases. However, TLR7 mutations can also be inherited, as evidenced by a report of a multiplex family where the F507S variant was maternally transmitted to two affected siblings (2). Recently, the genetic spectrum of the disease expanded with the identification of a somatic TLR7 GOF mutation (F506S) in a patient with early-onset SLE, indicating that somatic mosaicism should also be considered in the etiology of this condition (5).

Clinical manifestations were dominated by SLE with frequent neurological complications and thrombocytopenia. AGS-like features with cerebral calcifications were documented in neonatal-onset cases, while F507 codon variants were linked to severe disease. Notably, our patient presented with severe hypertriglyceridemia (3,270 mg/dl), representing the first reported metabolic manifestation of TLR7 GOF disease. This finding is mechanistically plausible, given that chronic use of external interferon-α induces hypertriglyceridemia through the suppression of hepatic triglyceride lipase and lipoprotein lipase activity. There are also reports of autoantibodies against lipoprotein lipase in SLE, which can lead to hyperlipidemia. Additionally, the potential contribution of therapeutic interventions to the observed hypertriglyceridemia cannot be excluded. While our findings suggest a mechanistic link, further research is required to evaluate the prevalence of hypertriglyceridemia in broader cohorts of patients with type I interferonopathies.

The clinical responses to treatment provided valuable therapeutic insights. While four out of five patients treated with Janus kinase (JAK) inhibitors showed improved responses, conventional immunosuppressives showed limited sustained efficacy among the patients. In our case, ruxolitinib treatment led to rapid normalization of hypertriglyceridemia, but only partial amelioration of thrombocytopenia (platelet counts 100–120 × 10^9^/L) with persisting hypocomplementemia. This suggests that higher doses of ruxolitinib, or more specific upstream blockade using novel TLR7 inhibitors, may be required to achieve complete immunological and hematological remission.

The identification of *TLR7* GOF mutations offers a convincing scientific rationale for direct TLR7 inhibition as a targeted therapy. Enpatoran, a dual TLR7/8 inhibitor, has demonstrated encouraging preliminary results in clinical trials for SLE.

Our patient exhibits a phenotypically unique F507S presentation in terms of neurologic symptoms. Notably, her neurological manifestations were subtle and initially obscured by the severity of the refractory thrombocytopenia and hypertriglyceridemia. Even though the disease started early, she avoided severe neurological sequelae, exhibiting only modest developmental delay and white matter lesions, in contrast to three previously reported F507S individuals who experienced devastating AGS-like neurological consequences and cerebral vasculitis (2). This phenotypic heterogeneity suggests that early targeted therapy may prevent severe complications. Nevertheless, given the patient’s young age, the possibility of future progression toward more severe AGS-like neurological degradation cannot be excluded, underscoring the need for long-term monitoring.

In conclusion, we report a new patient with *TLR7* GOF mutation, representing the fourth identified case with the F507S variant. She shows the first metabolic manifestation (severe hypertriglyceridemia) and demonstrates a therapeutic response to JAK inhibition. JAK inhibitors produce superior clinical responses, supporting their use as first-line therapy. TLR7 inhibitors are becoming more widely available, which may lead to more targeted intervention. Considering the expanding number of genes underlying such conditions, next-generation gene panel or whole-exome sequencing offer significant benefit for the diagnosis of autoimmune cytopenia or in patients with early-onset SLE. Early detection is crucial for precision medicine approaches in this rare cause of early-onset autoimmunity and interferonopathy.

## Data Availability

National Center for Biotechnology Information ClinVar entry [VCV004086267.1] is available at https://www.ncbi.nlm.nih.gov/clinvar/variation/VCV004086267.1 (accessed November 20, 2025).
